# The Effect of Affective Context on Visuocortical Processing of Neutral Faces in Social Anxiety

**DOI:** 10.3389/fpsyg.2015.01824

**Published:** 2015-11-30

**Authors:** Matthias J. Wieser, David A. Moscovitch

**Affiliations:** ^1^Department of Psychology, University of WürzburgWürzburg, Germany; ^2^Department of Psychology and Centre for Mental Health Research, University of WaterlooWaterloo, Canada

**Keywords:** social anxiety, face processing, context effects, ERPs (Event-Related Potentials), EEG/ERP

## Abstract

It has been demonstrated that verbal context information alters the neural processing of ambiguous faces such as faces with no apparent facial expression. In social anxiety, neutral faces may be implicitly threatening for socially anxious individuals due to their ambiguous nature, but even more so if these neutral faces are put in self-referential negative contexts. Therefore, we measured event-related brain potentials (ERPs) in response to neutral faces which were preceded by affective verbal information (negative, neutral, positive). Participants with low social anxiety (LSA; *n* = 23) and high social anxiety (HSA; *n* = 21) were asked to watch and rate valence and arousal of the respective faces while continuous EEG was recorded. ERP analysis revealed that HSA showed elevated P100 amplitudes in response to faces, but reduced structural encoding of faces as indexed by reduced N170 amplitudes. In general, affective context led to an enhanced early posterior negativity (EPN) for negative compared to neutral facial expressions. Moreover, HSA compared to LSA showed enhanced late positive potentials (LPP) to negatively contextualized faces, whereas in LSA this effect was found for faces in positive contexts. Also, HSA rated faces in negative contexts as more negative compared to LSA. These results point at enhanced vigilance for neutral faces regardless of context in HSA, while structural encoding seems to be diminished (avoidance). Interestingly, later components of sustained processing (LPP) indicate that LSA show enhanced visuocortical processing for faces in positive contexts (happy bias), whereas this seems to be the case for negatively contextualized faces in HSA (threat bias). Finally, our results add further new evidence that top-down information in interaction with individual anxiety levels can influence early-stage aspects of visual perception.

## Introduction

Social anxiety disorder (SAD) is characterized by a “persistent fear of one or more social or performance situations in which the person is exposed to unfamiliar people or to possible scrutiny by others,” which leads to avoidance of, or intense anxiety or distress in these social situations (Diagnostic and Statistical Manual of Mental Disorders, DSM V; American Psychiatric Association, 2000). Cognitive models of SAD assume that cognitive biases in the processing of social information constitute important factors in the etiology and maintenance of this disorder (Beck et al., 1985; Clark and Wells, 1995; Bögels and Mansell, 2004; Schultz and Heimberg, 2008; Cisler and Koster, 2010; Morrison and Heimberg, 2013). Biased information processing of unambiguous (i.e., threatening) or ambiguous signals of negative evaluation by others has been repeatedly shown across studies of attention, memory, and interpretation (Heinrichs and Hofman, 2001; Bögels and Mansell, 2004; Morrison and Heimberg, 2013). Particularly the misinterpretation of neutral or affiliative social signals as threatening is likely to deepen distress and contribute to the maintenance of SAD (e.g., Clark and Wells, 1995; Rapee and Heimberg, 1997; Gilbert, 2001; Alden and Taylor, 2004). Just recently, a meta-analysis including 24 studies on self-reported emotional reactions to facial expressions confirmed that socially anxious individuals show lower approachability to all types of expressions and higher arousal in response to neutral expressions (Kivity and Huppert, 2015).

Results of studies using event-related brain potentials (ERPs) in order to unfold the time course of face processing in social anxiety have been decidedly mixed (for an extensive review, see Schulz et al., 2013). To investigate electro-cortical response to faces and facial expressions, the following ERP components are of interest: As early ERP components which indicate visual processing the occipital P100 and the face-specific occipito-temporal N170. The P100 has been found to be modulated by facial expressions (e.g., Wieser et al., 2012b), presumably reflecting selective attention to emotional compared to neutral facial expressions, as also found in non-emotional attention research (e.g., Hillyard and Münte, 1984; Hillyard and Anllo-Vento, 1998). Furthermore, the N170 an index of structural encoding of faces (Bentin et al., 1996), is also modified by facial expressions (for reviews, see Eimer, 2011; Vuilleumier and Righart, 2011), although the empirical evidence for an emotional modulation of the N170 is mixed and remains an issue of debate. Most relevant for the current research questions are the subsequent emotion-sensitive components such as the early posterior negativity (EPN), and the late positive potential (LPP) (for a review, see Hajcak et al., 2012). Both of these are enhanced in response to emotional faces (e.g., Mühlberger et al., 2009; Wieser et al., 2012a,b), and index relatively early (EPN) and sustained (LPP) motivated attention to salient stimuli (Schupp et al., 2004; Wieser et al., 2010, 2012a,b). With regards to the face-specific N170 component of the ERP, some studies reported no effect of social anxiety on N170 responses to angry faces (Kolassa et al., 2007, 2009; Mühlberger et al., 2009), whereas other studies found that highly socially anxious participants exhibited larger N170 amplitudes to angry faces than low-anxiety participants over right temporo-parietal sites (Kolassa and Miltner, 2006). Interestingly, some studies report overall reduced N170 amplitudes (or M170, the MEG equivalent) in response to faces in general in socially anxious individuals, suggesting reduced encoding of faces (Mueller et al., 2009; Riwkes et al., 2015). Earlier effects on the P100 such as an amplitude enhancement, which is an index of selective attention (Hillyard and Münte, 1984), indicate an early attentional bias for social stimuli (hypervigilance), which may not be dependent on threat content (Schulz et al., 2013). Some studies found enhanced P100 amplitudes in response to faces in general (Kolassa et al., 2009; Rossignol et al., 2012, 2013) or selectively in response to threatening (angry) faces (Helfinstein et al., 2008; Mueller et al., 2009; Rossignol et al., 2012). Emotion-related ERP components such as the EPN and the LPP were also observed to be modulated by social anxiety. The EPN as an index for early, motivated attention was found to be larger for angry (and fearful) faces in trait and state social anxiety (Mühlberger et al., 2009; Wieser et al., 2010). Some studies reported greater LPPs for threatening but also neutral faces (Moser et al., 2008; Mühlberger et al., 2009; Kujawa et al., 2015), and positive correlations between social anxiety and the P3 amplitude for angry but not for happy faces (Sewell et al., 2008). However, other studies did not report modulation of late positive ERP during the processing of facial expressions by individuals suffering from social anxiety (Rossignol et al., 2007; van Peer et al., 2010).

The findings of enhanced LPPs to neutral faces in social anxiety point at the notion that ambiguous faces or neutral faces may be more threatening for socially anxious compared to healthy controls, so far from being neutral. This assumption is also supported by fMRI studies showing enhanced amygdala activations to neutral faces (Birbaumer et al., 1998; Straube et al., 2005; Cooney et al., 2006; Gentili et al., 2008). On a behavioral level, it has been demonstrated that social anxiety is also characterized by an interpretation bias such that socially anxious individuals more often interpret neutral faces as being negative (Yoon et al., 2007; Yoon and Zinbarg, 2008). Recently, it also has been demonstrated that social anxiety is associated with an expectancy bias for new neutrals faces such that HSA individuals lack a positive expectancy bias toward new social partners (Bielak and Moscovitch, 2012).

While it seems clear that perception and interpretation of emotional facial expressions is modulated by contexts in general (Barrett et al., 2011; Wieser and Brosch, 2012; Hassin et al., 2013; Hess and Hareli, 2015), particularly when processing ambiguous faces, individuals rely on contextual information to evaluate faces and form first impressions. This may especially be true when feeling anxious: When participants saw ambiguous facial expressions and simultaneously, positive or negative contextual information appeared on the screen, participants with high state anxiety showed greater use of contextual information in the interpretation of the facial expressions (Richards et al., 2007). Recently, several studies have shown that also verbal contextual information given beforehand alters processing of ambiguous faces such a neutral or surprised faces (Kim et al., 2004; Schwarz et al., 2013; Wieser et al., 2014; Klein et al., 2015). In an fMRI study showing neutral faces which were put into negative, positive, and neutral contexts by preceding sentences, enhanced amygdala activity was found for faces put in negative contexts (Kim et al., 2004).

Adapting this paradigm with neutral faces and self-reference as an additional contextual variable (i.e., the sentences were either self-related for the observer vs. other-related), it was demonstrated that contextual information is able to modify brain activity in response to neutral faces which did not differ on perceptual level (Schwarz et al., 2013). Specifically, it was found that two brain areas were especially responsive to faces put in a self-referential context: the medial prefrontal cortex (mPFC) and the fusiform face area (FFA) in the fusiform gyrus. Whereas, mPFC is thought to play a role in processing of self-related information (e.g., Phan et al., 2004; Mitchell et al., 2005; Moran et al., 2006), activity in FFA is supposed to reflect face-specific activity and belongs to the core area of face processing (Haxby et al., 2000, 2002; Haxby and Gobbini, 2011). Rather intriguing, one has to bear in mind that the facial features for both categories are the same, which indicates a higher-order top-down influence of visual processing. In the same study, neutral faces put in a self-referential negative context were associated with enhanced activity in mPFC, which correlated with a measure of social anxiety (Schwarz et al., 2013). This is a first hint that contextual modulation of face processing may interact with individual levels of social anxiety, particularly so when the context is negatively framed. Recently, this paradigm was adapted to investigate ERP correlates of face processing (Wieser et al., 2014). Two important results emerged: (a) self-reference was found to modulate early and later stages of affective stimulus processing, namely the EPN and the LPP of the face-evoked ERP; and (b) affective valence of the context modulated early, but not later stages of affective face processing. These effects again occurred although faces *per se* did not differ perceptually. Affective ratings of the faces confirmed these findings. Altogether, these results demonstrate on both an electrocortical and behavioral level that contextual information modifies early visual perception in a top-down manner.

In the present study, we aimed at further examining how individual levels of social anxiety influence above-mentioned contextual modulation of neutral face processing. In addition to a replication of above-mentioned findings from behavioral studies, we sought to extend these findings to the neural level by using ERP methodology. As the fMRI results reported by Schwarz et al. (2013) suggest that the influence of social anxiety to be greatest for self-referential negative context information, we only investigated self-referential affective contexts (negative, positive, neutral) in this study. Based on previous findings showing biased processing of negative facial expressions (Staugaard, 2010; Gilboa-Schechtman and Shachar-Lavie, 2013), we assumed that individuals with high social anxiety would exhibit enhanced responding to negatively contextualized faces. This should be observable in affective ratings (higher arousal, more negative valence), and emotional components of the ERP (EPN, LPP). Based on previous findings, we also assumed that high social anxiety might show enhanced P100 amplitudes to faces in general as an index for hypervigilance. With regard to the face-selective N170 component, two alternative hypotheses were to be evaluated: N170 amplitudes could be enhanced in HSA selectively for negatively contextualized faces as previously observed for angry faces (e.g., Kolassa and Miltner, 2006), but also diminished as an index for perceptual avoidance or more superficial processing of faces (Mueller et al., 2009).

## Methods

### Participants

Participants were undergraduate students at the University of Würzburg without any past or present psychiatric diagnosis (self-report), who were paid or received course credit for participation. Over 700 students filled in a pre-screening questionnaire consisting of five items (Supplementary Table 1) based on the DSM-IV criteria for social phobia (American Psychiatric Association, 2000), on a five-point Likert scale (0 = “Strongly disagree” to 4 = “Strongly agree”), such that a maximum of 20 points could be achieved. Based on the distribution of total scores, we aimed at inviting the upper 30% and the lower 10–40% to participate. Thus, participants scoring from 4 to 7 points were classified as low (LSA) and participants scoring above 12 points were classified as high socially anxious (HSA). Overall, 26 HSA and 24 LSA participants were invited to take part in the study. One LSA participant had to be excluded due to excessive artifacts in the EEG, and three HSA participants were excluded due to self-reported depression and/or abnormal BDI scores (>22), so that 47 participants (HSA: *n* = 24; LSA: *n* = 23) were included in the statistical analysis.

Mean questionnaire and age scores are given in Table 1. Groups did not differ in terms of age [*t*_(42)_ = 1.61, *p* = 0.12] and sex ratio [HSA: 19 women; LSA: 19 women; $\chi_{\left(1,\;N=44\right)}^{2}=0.577$, *p* = 0.38]. To ensure that the screening was successful, participants completed the German version of the Social Phobia and Anxiety Inventory (SPAI; Turner et al., 1989; Fydrich, 2002). As expected, significant group differences were found in the total scores of the SPAI, *t*_(44)_ = 7.09, *p* < 0.001; HSA: *M* = 100.54, *SD* = 23.90; LSA: *M* = 53.87, *SD* = 19.73. Before the experimental task, participants also completed a socio-demographic questionnaire, the State-Trait Anxiety Inventory (STAI; Spielberger et al., 1970; Laux et al., 1981), the Beck Depression Inventory (BDI; Beck et al., 1961; Hautzinger et al., 2006), and the Positive and Negative Affect Scale (PANAS; Watson et al., 1988; Krohne et al., 1996). Groups did not differ in PANAS and state anxiety (STAI-S), but HSAs scored significantly higher on measures of trait anxiety (STAI-T) and depression (BDI), *t*_(42)_ = 4.99, *p* < 0.001, and *t*_(42)_ = 2.45, *p* = 0.018.

**Table 1:** **Mean questionnaire and age scores for high socially anxious (HSA) and (LSA) participants**.

**Variable**	**HSA (*n* = 21)**		**LSA (*n* = 23)**		***t***	***p***
	***M***	***SD***	***M***	***SD***		
Age	20.81	1.86	22.17	3.43	1.62	0.114
SPAI	100.54	23.90	53.87	19.74	7.09	<**0.001**
STAI State	40.38	7.24	36.09	7.24	2.57	0.070
STAI Trait	47.00	10.63	33.83	6.59	4.99	<**0.001**
BDI	8.05	5.55	4.70	3.35	2.45	**0.018**
PANAS_PAM^*^	27.81	4.90	28.14	4.83	0.22	0.827
PANAS_NA^*^	13.04	4.14	13.10	3.52	0.37	0.971

*SPAI, Social Phobia and Anxiety Inventory; STAI, State-Trait Anxiety Inventory; BDI, Beck Depression Inventory; PANAS, Positive and Negative Affect Scale (PA, positive affect; NA, negative affect)*.

* *n = 22 for the LSA group due to one participant missed filling in the PANAS questionnaire. Significant p-values are given in bold*.

Experimental procedures were approved by the institutional review board of the University of Würzburg, and all participants provided informed consent. All participants of the final sample were free of any neurological or psychiatric disorder (self-report) and had normal or corrected-to-normal vision.

### Stimulus materials

Thirty-six pictures (18 females) were selected from the Radboud Faces Database (Langner et al., 2010), all showing neutral facial expressions in frontal view. Pictures were selected based on normative ratings with regards to best percentage of agreement on emotion categorization and mean genuineness (Langner et al., 2010). Pictures were converted to gray-scale, and the contrast was approximated by calculating the variance, which was standardized across all the Radboud faces in order to minimize physical differences.

The paradigm was taken from a previous study from our lab (Wieser et al., 2014): For the context stimuli, 36 sentences were created, varying in terms of valence (positive, neutral, and negative), resulting in six sentences per category (for examples, see Wieser et al., 2014). In order to minimize grammatical differences or differences in word length between sentences, all sentences were of the same grammatical structure. Moreover, each sentence of each category contained six words. In contrast to the previous study, only self-referential sentences were used.

### Procedure

**Figure 1:**
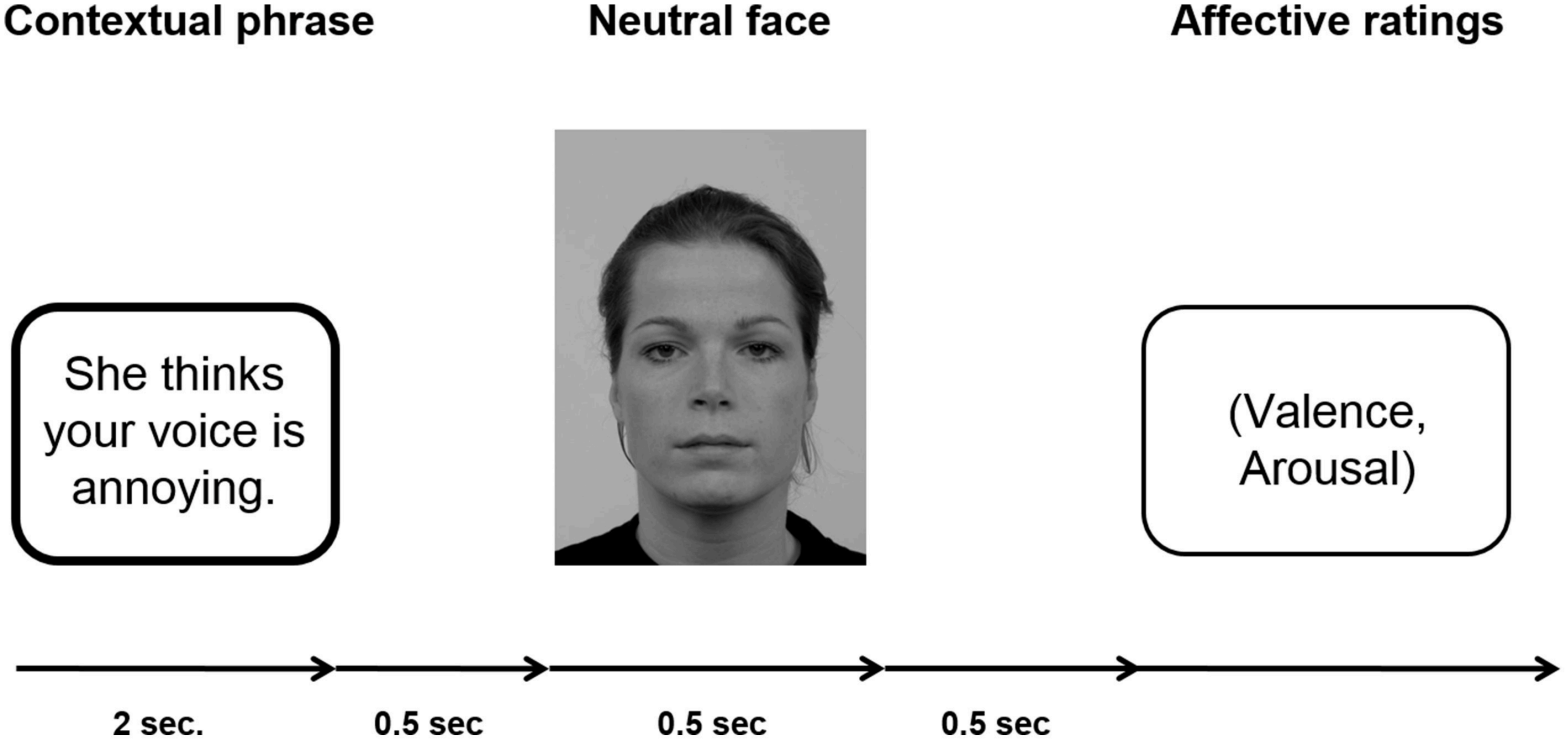
**Schematic of an experimental trial**. A fixation cross was shown during inter-trial interval (ITI), which lasted randomly 2 and 3 s.

Participants passively viewed sentences and neutral facial expressions according to the paradigm established by Wieser et al. (2014). For an example of an experimental trial see Figure 1.

Each sentence (positive, negative, neutral) was presented six times, three times with a male personal pronoun and three times with a female personal pronoun beginning the sentence. Consequently, each individual face was shown six times within a context category with different sentences. One set of three male and three female faces was assigned to positive sentences, another set of three male and three female faces was assigned to negative sentences, and the last set of three male and three female faces was assigned to neutral sentences. This assignment of picture sets to specific context valences was counterbalanced across participants to ensure that differences in the ERPs were not caused by intrinsic features of the faces. Overall, per session 72 trials per condition were presented (three male and three female faces repeated six times with the respective sentences) resulting in a total of 216 trials. In each trial, the sentence was presented for 2 s, after which with a gap of 500 ms a face was presented for 500 ms. After each trial, participants were asked to rate the respective face in terms of valence (−4 = very negative to +4 = very positive) and arousal (1 = not arousing at all to 9 = very arousing). The ratings scales were presented on the screen and the participants were asked to key in the respective number on a keyboard in front of them. Note that the valence scale −4 to +4 was stored as values ranging from 1 to 9. There was no time limit for the rating response. The ITI in which a fixation cross was presented, randomly varied between 2000 and 3000 ms. Presentation of the stimuli was controlled by presentation software (Neurobehavioral Systems, Inc., Albany, CA, USA), the pictures were shown on a 21-inch CRT-monitor (60 Hz refresh rate) located ~100 cm in front of the participant. Participants were instructed to keep their eyes comfortably focused on the center of the screen and to simply view the sentences and pictures, and rate the faces afterwards.

### EEG recording and data reduction

Brain and ocular scalp potentials were measured with a 128-channels geodesic sensor net (Electrical Geodesics, Inc., Eugene, OR, USA), on-line bandpass filtered from 0.1 to 100 Hz, and sampled at 250 Hz using Netstation acquisition software and EGI amplifiers. Electrode impedance was kept below 50 kΩ, as recommended for this type of high-impedance EEG amplifier. Data were recorded continuously with the vertex sensor as reference electrode. Continuous EEG data were low-pass filtered at 35 Hz using a zero-phase forward and reverse digital filter before stimulus-synchronized epochs were extracted from 200 ms pre-stimulus onset (face) to 800 ms post-stimulus onset and baseline-corrected (−100 ms). Preprocessing and artifact rejection were performed according to Junghöfer et al. (2000) using EMEGs software (Peyk et al., 2011). Off-line, data were re-referenced to an average reference. Afterwards, epochs were averaged for each participant and each experimental condition. ERP components were quantified on the basis of peak or mean amplitudes calculated over time windows defined on the basis of visual inspection and the literature (e.g., Wieser et al., 2010, 2014). The P100 component was analyzed as peak amplitude between 104 and 128 ms over right and left occipital electrode clusters including electrode O1 (EGI sensors 69, 70, 73, 74) and electrode O2 (EGI sensors 82, 83, 88, 89). For the N170 component, which reflects the early perceptual encoding stage of face processing, the peak amplitude was quantified between 152 and 182 ms after picture onset at lateral temporo-occipital clusters including electrodes P7 (EGI sensors 57, 58, 59, 63, 64, 65, 68, 69) and P8 (EGI sensors: 89, 90, 91, 94, 95, 96, 99, 100). The EPN was analyzed as an index of selective attention processes. It was scored as mean activity from 260 to 320 ms from a medial occipital cluster including Oz (EGI sensors 69, 70, 73, 74, 75, 81, 83, 88, 89). The LPP was analyzed (mean activity from 400 to 600 ms after face onset) as an index of sustained motivated attention across a central-parietal cluster (EGI sensors, 52, 53, 54, 55, 60, 61, 62, 67, 72, 77, 78, 79, 85, 86, 92) clusters.

### Statistical analysis

ERP measures as well as valence and arousal ratings were subjected to separate repeated-measures ANOVAs containing the within-subject factors Contextual Valence (negative vs. positive vs. neutral), and the between-subject factor Group (LSA vs. HSA). ANOVAs for lateralized ERPs (P100, N170) additionally contained the within-subjects factor hemisphere (left vs. right). If necessary, Greenhouse–Geisser correction of degrees of freedom (GG-ε) was applied. A significance level of 0.05 was used for all analyses. For all analyses, the uncorrected degrees of freedom, the corrected *p*-values, the GG-ε and the partial η^2^ ($\eta_{p}^{2}$) are reported (Picton et al., 2000).

## Results

### Event-related brain potentials (ERPs) in response to contextualized faces

#### P100

**Figure 2:**
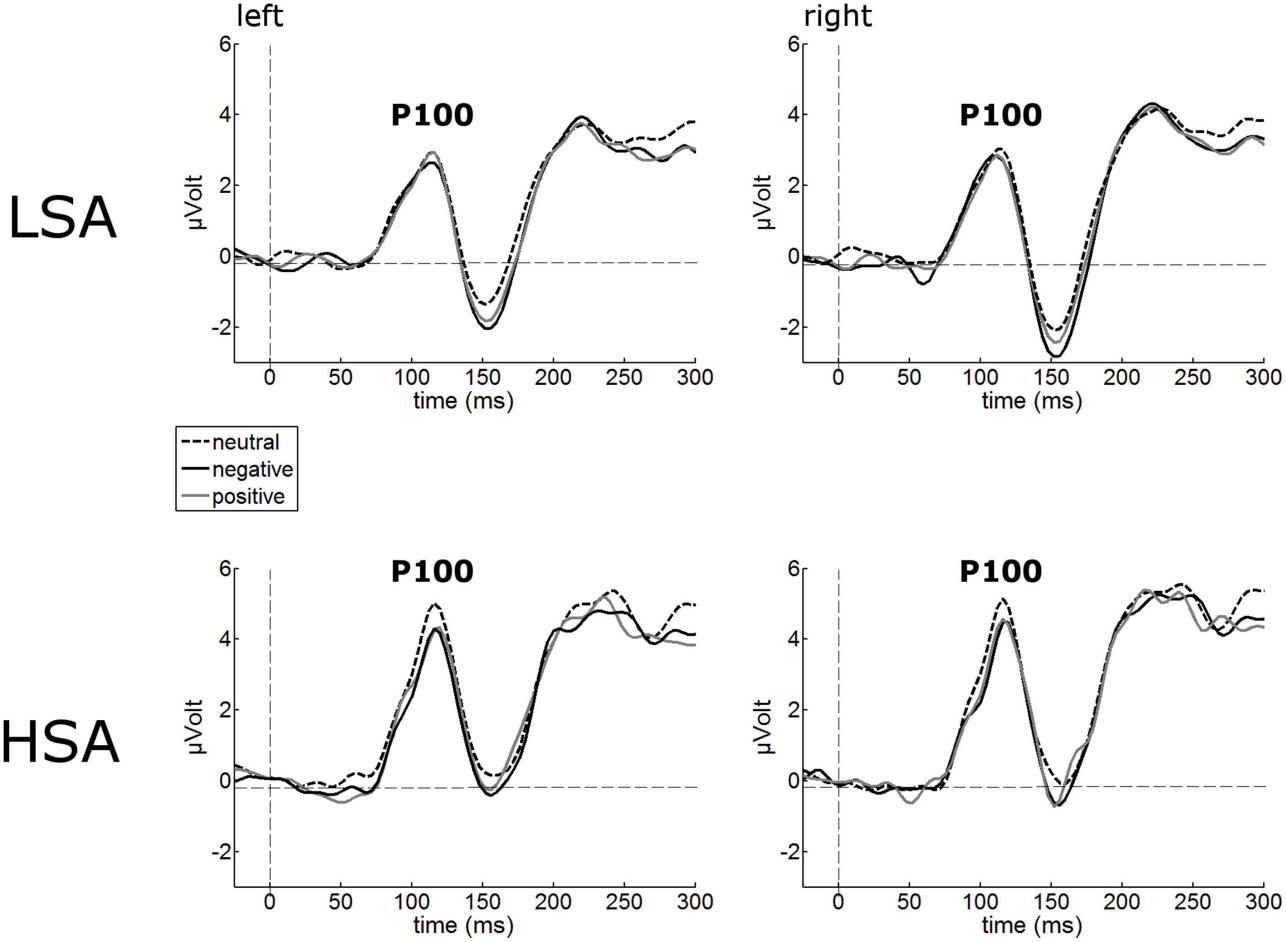
**Illustration of the P100 component averaged across left and right occipital electrode clusters per experimental group (HSA vs. LSA) for negatively, neutrally, and positively contextualized faces**. Overall, P100 amplitudes are enhanced in HSA compared to LSA.

The P100 of the face-evoked ERP did not show any effects of contextual valence. Also, no hemispheric differences were observed (Figure 2). However, a main effect of group was observed, *F*_(1, 42)_ = 7.24, *p* = 0.010, $\eta_{p}^{2}=0.147$, indicating larger P100 amplitudes in HSA (*M* = 5.05 μV, *SD* = 1.92) compared to LSA (*M* = 3.59 μV, *SD* = 1.68) in response to all faces, as expected from previous research.

#### N170

**Figure 3:**
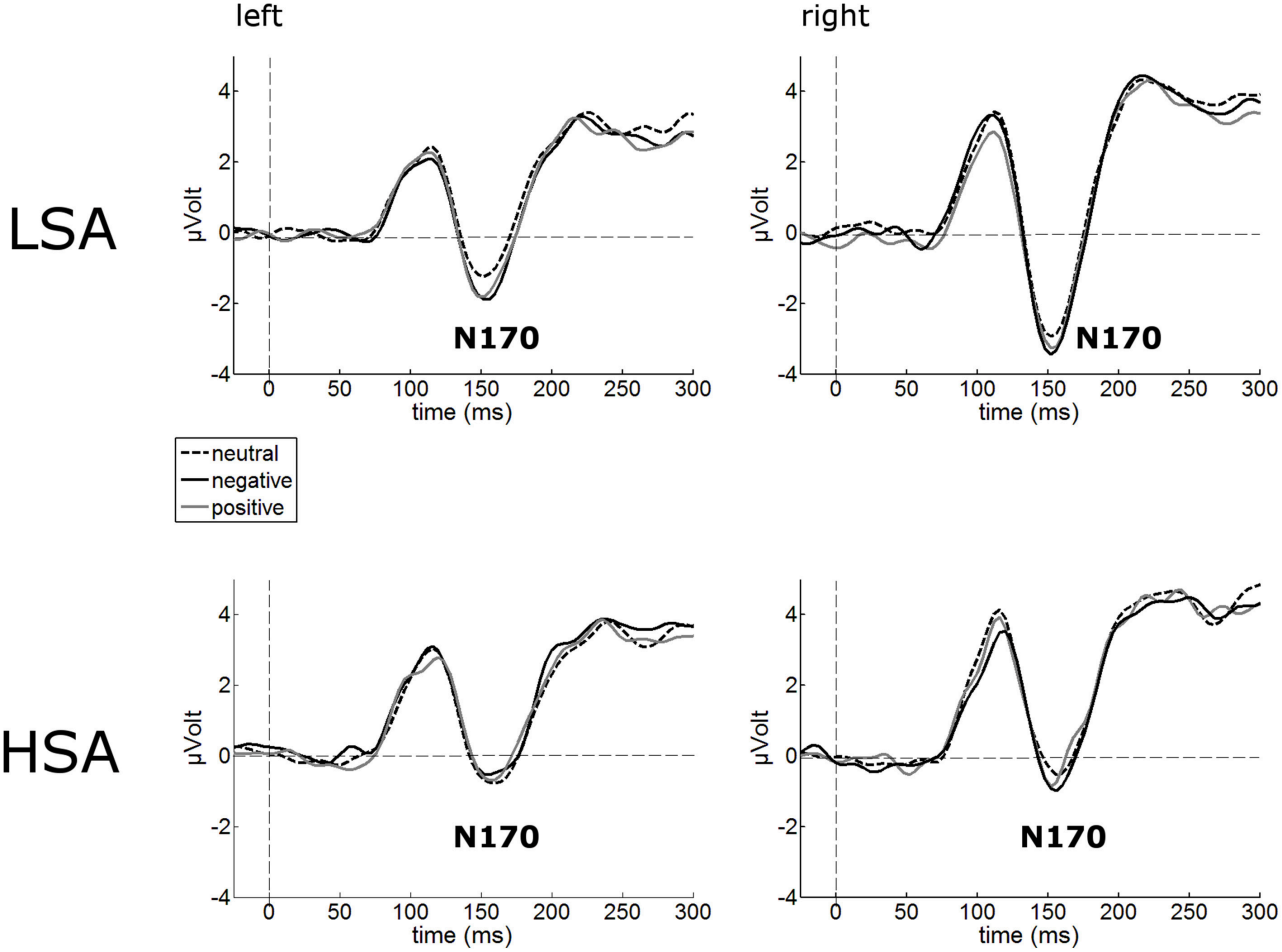
**Illustration of the N170 component averaged across left and right occipital electrode clusters per experimental group (HSA vs. LSA) for negatively, neutrally, and positively contextualized faces**. Overall, N170 amplitudes are diminished in HSA compared to LSA.

The N170 amplitudes of the face-evoked ERP were not modulated by contextual valence (Figure 3). Interestingly, N170 amplitudes were generally reduced in HSA (*M* = −1.22 μV, *SD* = 2.80) compared to LSA (*M* = −2.94 μV, *SD* = 2.67), *F*_(1, 42)_ = 4.32, *p* = 0.044, $\eta_{p}^{2}=0.093$ (Figure 3).

#### Early posterior negativity (EPN)

**Figure 4:**
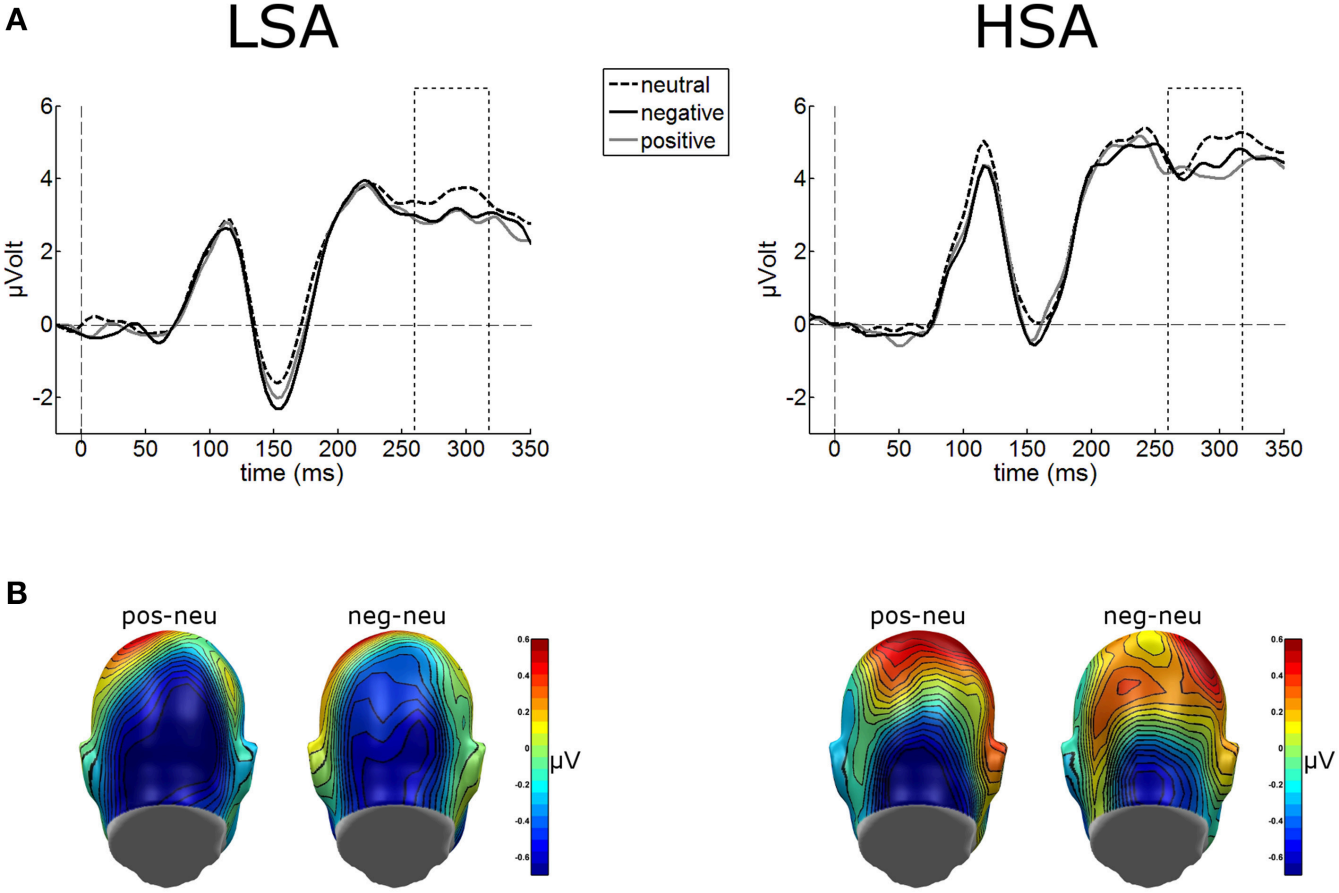
**(A)** Illustration of the EPN component (260–320 ms, see hatched box) averaged across medial occipital electrode cluster for both groups. **(B)** On a back view of the model head the scalp potential map of the difference waves “negative-neutral” and “positive-neutral” are given. No group differences emerged, but enhanced EPN was observed for negative and positive contextualized faces.

Cortical processing of neutral faces differed significantly in the EPN time window depending on verbal context presentation. For the mean EPN amplitudes (260–320 ms), a significant main effect of contextual valence was observed as expected, *F*_(2, 84)_ = 3.48, *p* = 0.043, $\eta_{p}^{2}=0.077$. Faces put in a negative context elicited an increased relative negativity as compared to faces put in neutral contexts, *F*_(1, 42)_ = 4.71, *p* = 0.036, $\eta_{p}^{2}=0.10$ (Figure 4). The same effect was found for faces in positive compared to neutral contexts, *F*_(1, 42)_ = 4.64, *p* = 0.037, $\eta_{p}^{2}=0.10$. No other modulations were observed.

#### Late positive potential (LPP)

**Figure 5:**
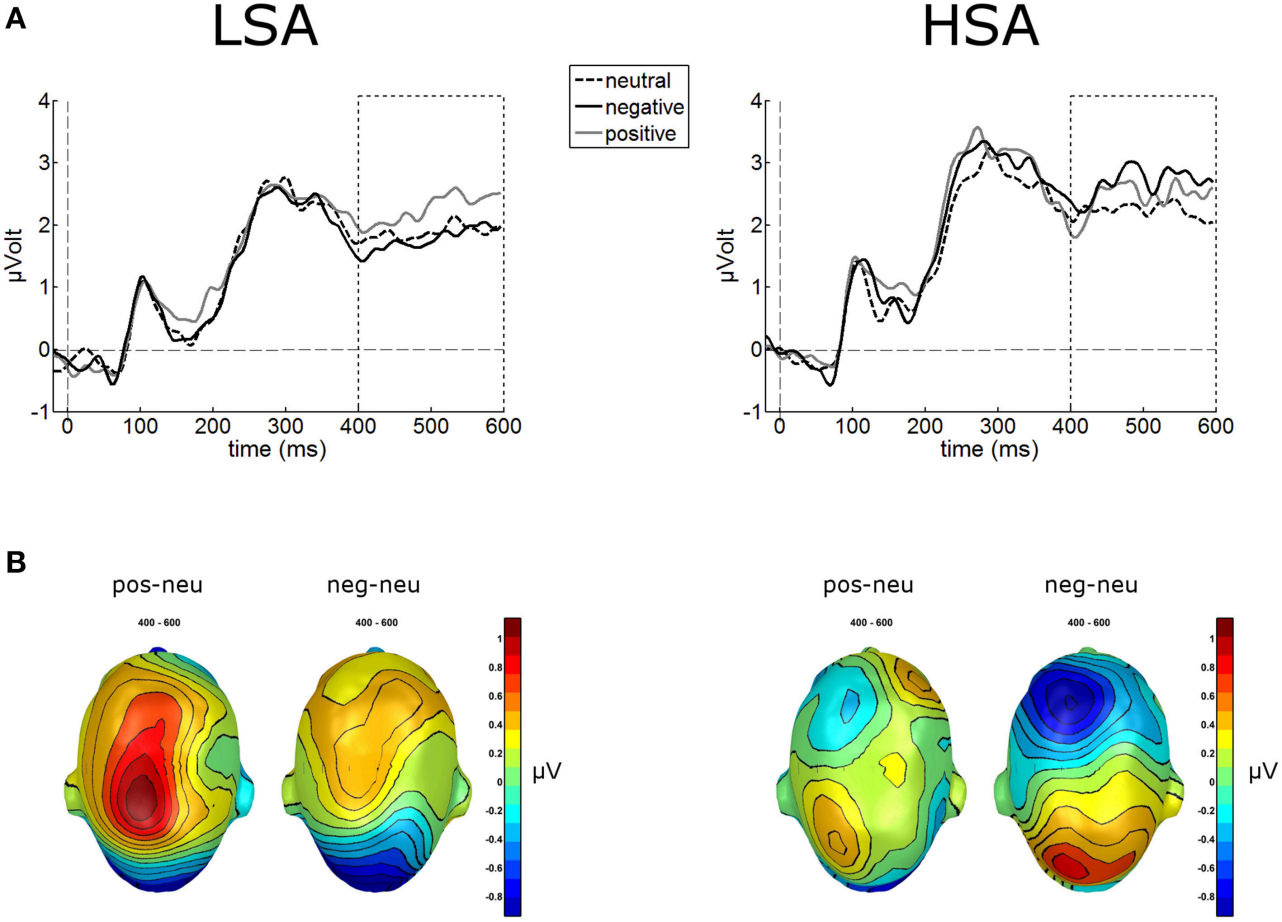
**Illustration of the LPP component averaged across medial-central sensor cluster for the three contextual conditions per group**. **(A)** Enhanced LPP amplitudes were observed for positively contextualized compared to neutrally contextualized faces in LSA, but for negatively contextualized faces in HSA. **(B)** Scalp potential maps of the difference waves “negative-neutral” and “positive-neutral” for the LPP component are given on a top view of the model head.

The waveform analyses revealed highly significant modulations of the LPP as a function of contextual valence and group, *F*_(2, 84)_ = 3.32, *p* = 0.041, $\eta_{p}^{2}=0.073$ (see Figure 5). *Post-hoc* simple *t*-tests performed for each group revealed that in LSA, faces in positive contexts elicited enhanced LPP amplitudes compared to faces put in negative or neutral contexts, *t*_(22)_ = 2.53, *p* = 0.019, and *t*_(22)_ = 2.53, *p* = 0.019. As expected, in HSA faces in negative contexts elicited larger LPP amplitudes compared to faces in neutral contexts, *t*_(20)_ = 2.12, *p* = 0.046 (Figure 5).

### Affective ratings of faces

**Figure 6:**
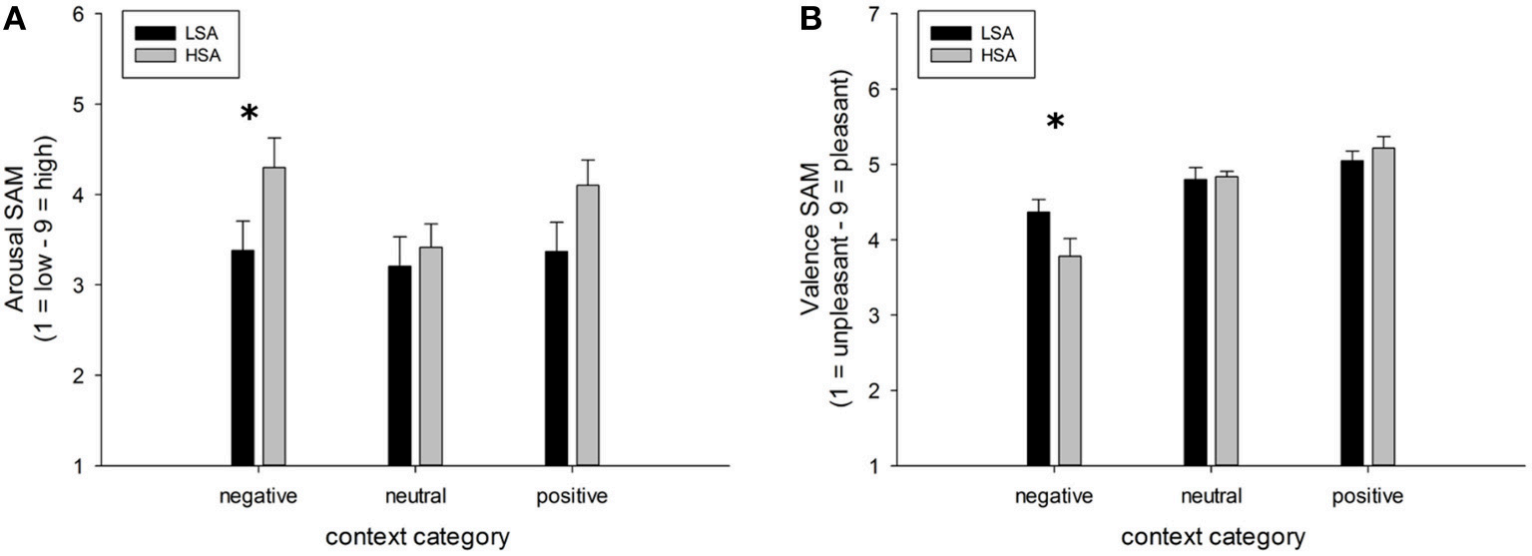
**Mean ratings (+SEM) of arousal (A) and valence (B) for faces in negative, neutral, and positive contexts, separated per group**. Group differences emerged for the negatively contextualized faces, only. ^*^Indicates *p*-values < 0.05.

A highly significant main effect of contextual valence was observed for arousal ratings of faces, *F*_(2, 84)_ = 7.74, *p* < 0.001, GG-ε = 0.81, $\eta_{p}^{2}=0.16$. This modulation was slightly differentially expressed in both groups, *F*_(2, 84)_ = 7.74, *p* = 0.053, $\eta_{p}^{2}=0.07$. (Figure 6A), mostly due to a tendency for HSA compared to LSA participants to rate faces in negative contexts to be more arousing, *t*_(42)_ = 1.98, *p* = 0.054.

For valence ratings of faces, a highly significant main effect of contextual valence was observed, *F*_(2, 84)_ = 27.48, *p* < 0.001, GG-ε = 0.60, $\eta_{p}^{2}=0.40$. However, this affective modulation was differentially expressed in both groups as there was a significant Group x Contextual valence interaction effect, *F*_(2, 84)_ = 3.78, *p* = 0.050, $\eta_{p}^{2}=0.08$. *Post-hoc* comparisons between groups revealed that faces in a negative context were rated as more negative by HSA compared to LSA participants, *t*_(42)_ = 2.04, *p* = 0.047, whereas no differences emerged between groups for faces in positive or neutral contexts (Figure 6B).

### Affective ratings of sentences

All the participants were asked to rate the sentences with regard to arousal and valence in a separate run after the main experiment (Table 2). Repeated-measures ANOVAs containing the within-subjects factor Contextual Valence (negative vs. neutral vs. positive) and the between-subjects factor Group were run on valence and arousal ratings separately. As expected, a significant main effect of contextual valence was observed for valence ratings, *F*_(2, 84)_ = 426.58, *p* < 0.001, $\eta_{p}^{2}=0.91$, with negative sentences being rated as more negative compared to neutral ones, *F*_(1, 42)_ = 410.89, *p* < 0.001, $\eta_{p}^{2}=0.91$, and positive sentences being rated as more positive compared to neutral ones, *F*_(1, 42)_ = 337.16, *p* < 0.001, $\eta_{p}^{2}=0.89$. A nearly significant Contextual Valence × Group interaction, *F*_(2, 84)_ = 3.71, *p* = 0.055, $\eta_{p}^{2}=0.81$, indicated that this effect was different in both groups. *Post-hoc* comparisons revealed that only for negative sentences, HSA showed significant more negative valence ratings compared to LSA, *t*_(42)_ = 2.43, *p* = 0.019.

**Table 2:** **Mean affective ratings + SD (valence and arousal) of sentences with contexts (negative, neutral, positive) in both groups (HSA, LSA)**.

**Contextual valence**	**HSA (*n* = 21)**		**LSA (*n* = 23)**	
	**Valence**	**Arousal**	**Valence**	**Arousal**
Negative	2.35 (0.58)	5.96 (1.69)	2.88 (0.82)	4.72 (2.08)
Neutral	5.22 (0.31)	2.02 (1.26)	5.07 (0.28)	2.23 (1.39)
Positive	7.41 (0.86)	5.63 (1.67)	7.12 (0.77)	4.87 (2.19)

For arousal ratings of the sentences, also a significant main effect of contextual valence was observed, *F*_(2, 82)_ = 118.24, *p* < 0.001, $\eta_{p}^{2}=0.74$, with negative and positive sentences being rated as more arousing compared to neutral ones, *F*_(1, 42)_ = 127.02, *p* < 0.001, $\eta_{p}^{2}=0.75$, and *F*_(1, 42)_ = 142.46, *p* < 0.001, $\eta_{p}^{2}=0.77$, respectively. Here, the interaction of group and contextual valence was highly significant, *F*_(2, 84)_ = 4.92, *p* = 0.01, GG-ε = 0.68, $\eta_{p}^{2}=0.11$. *Post-hoc* comparisons showed that HSA selectively rated negative sentences as more negative than LSA participants did, *t*_(42)_ = 2.16, *p* = 0.036, corroborating the findings in the valence ratings.

## Discussion

How does trait social anxiety influence the contextual modulation of neutral face processing? The present study investigated the possible association of social anxiety and the influence of affective context features on the evaluation and electrocortical processing of neutral human faces. To this end, participants high and low in social anxiety (HSA vs. LSA) viewed neutral facial expressions, which were preceded by phrases conveying contextual information about affective valence. Meanwhile, event-related brain potentials (ERPs) in response to the neutral face stimuli were recorded and affective ratings of these faces were obtained.

Results revealed main effects of contextual valence on early as well as later stages of electro-cortical affective stimulus processing (as indexed by EPN and LPP), which is in line with our previous findings where negative affective context was associated with enhanced early preferential processing as indexed by an emotional modulation of the EPN (Wieser et al., 2014). Interestingly, this modulation of face processing was differentially expressed in HSA compared to LSA at later stages of face processing (LPP). At this later stage, HSA showed enhanced processing of negatively contextualized compared to neutral faces, whereas for LSA highest LPP amplitudes were observed for positively contextualized faces. Affective ratings support these ERP findings, with higher arousal ratings for negative and positive compared to neutral contextualized faces. Selectively, HSA rated negatively contextualized faces as more arousing and more negative. At earlier stages of visuocortical face processing, two main effects of social anxiety were observed: HSA show hypervigilance for faces in general (enhanced P100 amplitudes), but reduced structural encoding of faces (diminished N170 amplitudes).

The enhanced P100 in HSA in response to faces is consistent with a plethora of studies in which HSA or patients with SAD showed increased amplitudes of the face-evoked P100 component (Kolassa et al., 2009; Mueller et al., 2009; Rossignol et al., 2012, 2013; Peschard et al., 2013). This effect was also observed when social anxiety was induced by a fear-of-public-speaking task (Wieser et al., 2010). As the P100 indexes selective attention (Hillyard and Anllo-Vento, 1998; Hillyard et al., 1998) and P100 enhancements were also found to threat-stimuli (Pourtois et al., 2005) and have therefore been assumed to indicate increased attention to threat (Vuilleumier and Pourtois, 2007), our findings again support the notion that social anxiety is characterized by initial hypervigilance to social stimuli and may indicate an early automatic attentional bias for social cues (Schulz et al., 2013).

Notwithstanding this early enhancement of face processing, HSA individuals in our study showed decreased N170 amplitudes in response to all face stimuli. This finding is also in line with some earlier studies, in which a decreased N170 (or its MEG equivalent, the M170) in response to faces was reported (Mueller et al., 2009; Riwkes et al., 2015). The N170/M170 face-selective component (Bentin et al., 1996) indexes structural encoding of faces, which includes a configurational analysis of whole faces. A reduced N170 in HSA might thus support the notion that an in-depth face analysis is avoided (Chen et al., 2002) or disrupted (Horley et al., 2003, 2004) in SAD. It has to be noted that others studies however report enhanced N170 amplitudes at least to some facial expressions such as anger (Kolassa and Miltner, 2006; Mühlberger et al., 2009). Most likely, these inconsistencies are a result of the different tasks employed in these studies (emotions were task relevant) or different types of facial stimuli (artificial vs. natural faces). Overall, our results are in line with earlier findings of studies indicating an attenuation of early neural components during face processing of individuals with high trait anxiety (e.g., Frenkel and Bar-Haim, 2011; Walentowska and Wronka, 2012). An interesting alternative explanation for the reduced N170 has been proposed by Riwkes et al. (2015). They assume that HSA use low-spatial frequency (LSF) information contained in faces differently such that they rely more on LSF information (connected to amygdala activation, see for example Vuilleumier et al., 2003) compared to high spatial frequency (HSF) information (connected to fusiform activation, see for example Vuilleumier et al., 2003), resulting in a reduced N/M170.

While no differences between groups were found at the ERP correlate of early emotional discrimination (EPN), HSA showed largest LPP amplitudes in response to negatively contextualized faces, whereas LSA showed a positivity bias in this ERP component. Thus, although it seems that contextual information may not influence early stages of face processing, HSA show sustained processing of especially negatively contextualized faces. This is in line with previous findings of elevated LPP amplitudes in response to negative faces in SAD (Moser et al., 2008; Mühlberger et al., 2009; Kujawa et al., 2015), which point at sustained attentional capture by negative facial expressions, a result also supported by recent flicker paradigms employing steady-state visual evoked potentials (ssVEP) technique (McTeague et al., 2011; Wieser et al., 2011, 2012c). However, one has to bear in mind that in the former studies the face stimuli were inherently negative (i.e., they contained negative facial expressions such as fear, anger, etc.), whereas, in our study the perceptual information in the faces was the same, namely void of any emotion. This shows that even contextual information, which is not present anymore during face processing, influences visual processing in a top-down manner depending on individual levels of social anxiety. The latter findings also corroborate the results from affective ratings in our study, where HSA tended to rate negatively contextualized faces as more arousing and more negative compared to LSA. Overall, these effects support for the first time on a cortical level the findings of HSAs to perceive self-relevant social contexts as being more threatening (see Moscovitch, 2009), so this provides an account of some of the neural mechanisms that may be involved in negative interpretation biases of ambiguous stimuli (see also Moscovitch and Hofmann, 2007).

As a limitation of the present study, we have to acknowledge that only a sub-clinical sample and not individuals clinically diagnosed with SAD were investigated. We assume that observed effects would be more pronounced in a clinical sample or there might be additional effects, e.g., it has to be clarified whether individuals with SAD show differences in the processing of contextualized neutral faces at earlier stages of face processing already. Furthermore, the use of individually tailored affective sentences containing the respective individual phobic cues would be necessary to identify how much these effects depend on the individual content of fear (Pergamin-Hight et al., 2015). Another potential issue relates to the fact that the differences observed in later stages of face processing might not only be driven by higher anxiety or arousal levels in HSA, but also by other features of social anxiety such as potential differences in hostility/aggression toward others and social situations (Kashdan and McKnight, 2010) or fear of positive or negative evaluation (Weeks and Howell, 2014).

Altogether, the present study shows that social anxiety may be characterized by two main biases in face processing even when the face itself does not carry affective information: *(a) a general attentional bias (hypervigilance), but reduced configural processing of faces; and (b) a selective enhancement of processing negatively contextualized faces, which is also reflected in subjective ratings*. Overall, the present findings together with previous results (Schwarz et al., 2013; Wieser et al., 2014; Klein et al., 2015) support the notion that face processing is highly context-dependent (Wieser and Brosch, 2012), and this may even be more relevant and pronounced with elevated levels of social anxiety. Further research may clarify to what extent HSA individuals rely on context information when they encounter emotional facial expressions and whether they could be trained to reappraise contextual information as a useful strategy for modifying attentional biases in social perception similar to attentional bias modification training (Beard et al., 2012) or re-appraisal of emotional stimuli (Moscovitch et al., 2012). Clearly, new research also needs to take into account other non-verbal social cues as potential contextual modulators of face processing in social anxiety (Bielak and Moscovitch, 2013; Gilboa-Schechtman and Shachar-Lavie, 2013).

## Funding

This publication was funded by the German Research Foundation (DFG) and the University of Wuerzburg in the funding program Open Access Publishing.

### Conflict of interest statement

The authors declare that the research was conducted in the absence of any commercial or financial relationships that could be construed as a potential conflict of interest.

## References

[B1] Alden L. E. Taylor C. T. (2004). Interpersonal processes in social phobia. Clin. Psychol. Rev. 24, 857–882. 10.1016/j.cpr.2004.07.006 15501559

[B2] American Psychiatric Association (2000). Diagnostic and Statistical Manual of Mental Disorders (DSM-IV). Washington, DC: American Psychiatric Publishing.

[B3] Barrett L. F. Mesquita B. Gendron M. (2011). Context in emotion perception. Curr. Dir. Psychol. Sci. 20, 286–290. 10.1177/0963721411422522

[B4] Beard C. Sawyer A. T. Hofmann S. G. (2012). Efficacy of attention bias modification using threat and appetitive stimuli: a meta-analytic review. Behav. Ther. 43, 724–740. 10.1016/j.beth.2012.01.002 23046776 PMC3494088

[B5] Beck A. T. Emery G. Greenberg R. L. (1985). Anxiety Disorders and Phobias: A Cognitive Perspective. New York, NY: Basic Books.

[B6] Beck A. T. Ward C. H. Mendelson M. Mock J. Erbaugh J. (1961). An inventory for measuring depression. Arch. Gen. Psychiatry 4, 561–571. 10.1001/archpsyc.1961.01710120031004 13688369

[B7] Bentin S. Allison T. Puce A. Perez E. McCarthy G. (1996). Electrophysiological studies of face perception in humans. J. Cogn. Neurosci. 8, 551–565. 10.1162/jocn.1996.8.6.551 20740065 PMC2927138

[B8] Bielak T. Moscovitch D. A. (2012). Friend or foe? Memory and expectancy biases for faces in social anxiety. J. Exp. Psychopathol. 3, 42–61. 10.5127/jep.1971126481612

[B9] Bielak T. Moscovitch D. A. (2013). How do I measure up? The impact of observable signs of anxiety and confidence on interpersonal evaluations in social anxiety. Cogn. Ther. Res. 37, 266–276. 10.1007/s10608-012-9473-4

[B10] Birbaumer N. Grodd W. Diedrich O. Klose U. Erb M. Lotze M.. (1998). fMRI reveals amygdala activation to human faces in social phobics. Neuroreport 9, 1223–1226. 10.1097/00001756-199804200-00048 9601698

[B11] Bögels S. M. Mansell W. (2004). Attention processes in the maintenance and treatment of social phobia: hypervigilance, avoidance and self-focused attention. Clin. Psychol. Rev. 24, 827–856. 10.1016/j.cpr.2004.06.005 15501558

[B12] Chen Y. P. Ehlers A. Clark D. M. Mansell W. (2002). Patients with generalized social phobia direct their attention away from faces. Behav. Res. Ther. 40, 677–687. 10.1016/S0005-7967(01)00086-9 12051486

[B13] Cisler J. M. Koster E. H. W. (2010). Mechanisms of attentional biases towards threat in anxiety disorders: an integrative review. Clin. Psychol. Rev. 30, 203–216. 10.1016/j.cpr.2009.11.003 20005616 PMC2814889

[B14] Clark D. M. Wells A. (1995). A cognitive model of social phobia, in Social Phobia: Diagnosis, Assessment, and Treatment, ed Heimberg R. G. (New York, NY: Guilford Press), 69–93.

[B15] Cooney R. E. Atlas L. Y. Joormann J. Eugène F. Gotlib I. H. (2006). Amygdala activation in the processing of neutral faces in social anxiety disorder: is neutral really neutral? Psychiatry Res. 148, 55–59. 10.1016/j.pscychresns.2006.05.003 17030117

[B16] Eimer M. (2011). The face-sensitive N170 component of the event-related brain potential, in The Oxford Handbook of Face Perception, eds Calder A. Rhodes G. Johnson M. Haxby J. (Oxford: Oxford University Press), 329–344.

[B17] Frenkel T. I. Bar-Haim Y. (2011). Neural activation during the processing of ambiguous fearful facial expressions: an ERP study in anxious and nonanxious individuals. Biol. Psychol. 88, 188–195. 10.1016/j.biopsycho.2011.08.001 21846487

[B18] Fydrich T. (2002). SPAI - Soziale Phobie und Angst Inventar, in Diagnostische Verfahren in der Psychotherapie, eds Brähler E. Schumacher J. Strauß B. (Göttingen: Hogrefe), 335–338.

[B19] Gentili C. Gobbini M. I. Ricciardi E. Vanello N. Pietrini P. Haxby J. V.. (2008). Differential modulation of neural activity throughout the distributed neural system for face perception in patients with Social Phobia and healthy subjects. Brain Res. Bull. 77, 286–292. 10.1016/j.brainresbull.2008.08.003 18771714

[B20] Gilbert P. (2001). Evolution and social anxiety. The role of attraction, social competition, and social hierarchies. Psychiatr. Clin. North Am. 24, 723–751. 10.1016/S0193-953X(05)70260-4 11723630

[B21] Gilboa-Schechtman E. Shachar-Lavie I. (2013). More than a face: a unified theoretical perspective on nonverbal social cue processing in social anxiety. Front. Hum. Neurosci. 7:904. 10.3389/fnhum.2013.00904 24427129 PMC3876460

[B22] Hajcak G. Weinberg A. Macnamara A. Foti D. (2012). ERPs and the study of emotion, in Oxford Handbook of ERP Components, eds Luck S. J. Kappenman E. S. (New York, NY: Oxford University Press), 441–474.

[B23] Hassin R. R. Aviezer H. Bentin S. (2013). Inherently ambiguous: facial expressions of emotions, in context. Emot. Rev. 5, 60–65. 10.1177/1754073912451331

[B24] Hautzinger M. Keller F. Kühner C. (2006). Beck Depressionsinventar – II. Frankfurt/Main: Hartcourt.

[B25] Haxby J. V. Gobbini M. I. (2011). Distributed neural systems for face perception, in The Oxford Handbook of Face Perception, eds Calder A. J. Rhodes G. Johnson M. H. Haxby J. V. (New York, NY: Oxford University Press), 93–110.

[B26] Haxby J. V. Hoffman E. A. Gobbini M. I. (2000). The distributed human neural system for face perception. Trends Cogn. Sci. (Regul. Ed). 4, 223–232. 10.1016/S1364-6613(00)01482-0 10827445

[B27] Haxby J. V. Hoffman E. A. Gobbini M. I. (2002). Human neural systems for face recognition and social communication. Biol. Psychiatry 51, 59–67. 10.1016/S0006-3223(01)01330-0 11801231

[B28] Heinrichs N. Hofman S. G. (2001). Information processing in social phobia: a critical review. Clin. Psychol. Rev. 21, 751–770. 10.1016/S0272-7358(00)00067-2 11434229

[B29] Helfinstein S. M. White L. K. Bar-Haim Y. Fox N. A. (2008). Affective primes suppress attention bias to threat in socially anxious individuals. Behav. Res. Ther. 46, 799–810. 10.1016/j.brat.2008.03.011 18472088 PMC2527506

[B30] Hess U. Hareli S. (2015). The role of social context for the interpretation of emotional facial expressions, in Understanding Facial Expressions in Communication (Berlin: Springer), 119–141. 10.1007/978-81-322-1934-7_7

[B31] Hillyard S. A. Anllo-Vento L. (1998). Event-related brain potentials in the study of visual selective attention. Proc. Natl. Acad. Sci. U.S.A. 95, 781–787. 10.1073/pnas.95.3.781 9448241 PMC33798

[B32] Hillyard S. A. Münte T. F. (1984). Selective attention to color and location: an analysis with event-related brain potentials. Percept. Psychophys. 36, 185–198. 10.3758/BF03202679 6514528

[B33] Hillyard S. A. Vogel E. K. Luck S. J. (1998). Sensory gain control (amplification) as a mechanism of selective attention: electrophysiological and neuroimaging evidence. Philos. Trans. R. Soc. Lond. B Biol. Sci. 353, 1257–1270. 10.1098/rstb.1998.0281 9770220 PMC1692341

[B34] Horley K. Williams L. M. Gonsalvez C. Gordon E. (2003). Social phobics do not see eye to eye: a visual scanpath study of emotional expression processing. J. Anxiety Disord. 17, 33–44. 10.1016/S0887-6185(02)00180-9 12464287

[B35] Horley K. Williams L. M. Gonsalvez C. Gordon E. (2004). Face to face: visual scanpath evidence for abnormal processing of facial expressions in social phobia. Psychiatry Res. 127, 43–53. 10.1016/j.psychres.2004.02.016 15261704

[B36] Junghöfer M. Elbert T. Tucker D. M. Rockstroh B. (2000). Statistical control of artifacts in dense array EEG/MEG studies. Psychophysiology 37, 523–532. 10.1111/1469-8986.3740523 10934911

[B37] Kashdan T. B. McKnight P. E. (2010). The darker side of social anxiety: when aggressive impulsivity prevails over shy inhibition. Curr. Dir. Psychol. Sci. 19, 47–50. 10.1177/0963721409359280

[B38] Kim H. Somerville L. H. Johnstone T. Polis S. Alexander A. L. Shin L. M.. (2004). Contextual modulation of amygdala responsivity to surprised faces. J. Cogn. Neurosci. 16, 1730–1745. 10.1162/0898929042947865 15701225

[B39] Kivity Y. Huppert J. D. (2015). Emotional reactions to facial expressions in social anxiety: a meta-analysis of self-reports. Emot. Rev. 10.1177/1754073915594436. [Epub ahead of print].

[B40] Klein F. Iffland B. Schindler S. Wabnitz P. Neuner F. (2015). This person is saying bad things about you: the influence of physically and socially threatening context information on the processing of inherently neutral faces. Cogn. Affect. Behav. Neurosci. 15, 736–748. 10.3758/s13415-015-0361-8 25967930

[B41] Kolassa I.-T. Kolassa S. Bergmann S. Lauche R. Dilger S. Miltner W. H. R. (2009). Interpretive bias in social phobia: an ERP study with morphed emotional schematic faces. Cogn. Emot. 23, 69–95. 10.1080/02699930801940461

[B42] Kolassa I.-T. Kolassa S. Musial F. Miltner W. H. R. (2007). Event-related potentials to schematic faces in social phobia. Cogn. Emot. 21, 1721–1744. 10.1080/02699930701229189

[B43] Kolassa I.-T. Miltner W. H. R. (2006). Psychophysiological correlates of face processing in social phobia. Brain Res. 1118, 130–141. 10.1016/j.brainres.2006.08.019 16970928

[B44] Krohne H. W. Egloff B. Kohlmann C.-W. Tausch A. (1996). Untersuchungen mit einer deutschen version der “Positive and Negative Affect Schedule” (PANAS). Diagnostica 42, 139–156.

[B45] Kujawa A. MacNamara A. Fitzgerald K. D. Monk C. S. Phan K. L. (2015). Enhanced neural reactivity to threatening faces in anxious youth: evidence from event-related potentials. J. Abnorm. Child Psychol. 43, 1493–1501. 10.1007/s10802-015-0029-4 25943264 PMC4751035

[B46] Langner O. Dotsch R. Bijlstra G. Wigboldus D. H. J. Hawk S. T. van Knippenberg A. (2010). Presentation and validation of the Radboud Faces Database. Cogn. Emot. 24, 1377–1388. 10.1080/02699930903485076

[B47] Laux L. Glanzmann P. Schaffner P. Spielberger C. D. (1981). Das State-Trait-Angstinventar. Weinheim: Beltz Test.

[B48] McTeague L. M. Shumen J. R. Wieser M. J. Lang P. J. Keil A. (2011). Social vision: sustained perceptual enhancement of affective facial cues in social anxiety. Neuroimage 54, 1615–1624. 10.1016/j.neuroimage.2010.08.080 20832490 PMC3004773

[B49] Mitchell J. P. Banaji M. R. Macrae C. N. (2005). The link between social cognition and self-referential thought in the medial prefrontal cortex. J. Cogn. Neurosci. 17, 1306–1315. 10.1162/0898929055002418 16197685

[B50] Moran J. M. Macrae C. N. Heatherton T. F. Wyland C. L. Kelley W. M. (2006). Neuroanatomical evidence for distinct cognitive and affective components of self. J. Cogn. Neurosci. 18, 1586–1594. 10.1162/jocn.2006.18.9.1586 16989558

[B51] Morrison A. S. Heimberg R. G. (2013). Attentional control mediates the effect of social anxiety on positive affect. J. Anxiety Disord. 27, 56–67. 10.1016/j.janxdis.2012.10.002 23254261 PMC4598068

[B52] Moscovitch D. A. (2009). What is the core fear in social phobia? A new model to facilitate individualized case conceptualization and treatment. Cogn. Behav. Pract. 16, 123–134. 10.1016/j.cbpra.2008.04.002

[B53] Moscovitch D. A. Gavric D. L. Senn J. M. Santesso D. L. Miskovic V. Schmidt L. A. (2012). Changes in judgment biases and use of emotion regulation strategies during cognitive-behavioral therapy for social anxiety disorder: distinguishing treatment responders from nonresponders. Cognit. Ther. Res. 36, 261–271. 10.1007/s10608-011-9371-1

[B54] Moscovitch D. A. Hofmann S. G. (2007). When ambiguity hurts: social standards moderate self-appraisals in generalized social phobia. Behav. Res. Ther. 45, 1039–1052. 10.1016/j.brat.2006.07.008 16962994

[B55] Moser J. S. Huppert J. D. Duval E. Simons R. F. (2008). Face processing biases in social anxiety: an electrophysiological study. Biol. Psychol. 78, 93–103. 10.1016/j.biopsycho.2008.01.005 18353522

[B56] Mueller E. M. Hofmann S. G. Santesso D. L. Meuret A. E. Bitran S. Pizzagalli D. A. (2009). Electrophysiological evidence of attentional biases in social anxiety disorder. Psychol. Med. 39, 1141. 10.1017/s0033291708004820 19079826 PMC3204217

[B57] Mühlberger A. Wieser M. J. Hermann M. J. Weyers P. Tröger C. Pauli P. (2009). Early cortical processing of natural and artificial emotional faces differs between lower and higher socially anxious persons. J. Neural Transm. 116, 735–746. 10.1007/s00702-008-0108-6 18784899

[B58] Pergamin-Hight L. Naim R. Bakermans-Kranenburg M. J. van Ijzendoorn M. H. Bar-Haim Y. (2015). Content specificity of attention bias to threat in anxiety disorders: a meta-analysis. Clin. Psychol. Rev. 35, 10–18. 10.1016/j.cpr.2014.10.005 25462110

[B59] Peschard V. Philippot P. Joassin F. Rossignol M. (2013). The impact of the stimulus features and task instructions on facial processing in social anxiety: an ERP investigation. Biol. Psychol. 93, 88–96. 10.1016/j.biopsycho.2013.01.009 23384510

[B60] Peyk P. De Cesarei A. Junghöfer M. (2011). Electro Magneto Encephalography Software: overview and integration with other EEG/MEG toolboxes. Comput. Intell. Neurosci. 2011:861705. 10.1155/2011/861705 21577273 PMC3090751

[B61] Phan K. L. Wager T. D. Taylor S. F. Liberzon I. (2004). Functional neuroimaging studies of human emotions. CNS Spectr. 9, 258–266. 10.1017/S1092852900009196 15048050

[B62] Picton T. W. Bentin S. Berg P. Donchin E. Hillyard S. A. Johnson R. Jr.. (2000). Guidelines for using human event-related potentials to study cognition: recording standards and publication criteria. Psychophysiology 37, 127–152. 10.1111/1469-8986.3720127 10731765

[B63] Pourtois G. Thut G. Grave de Peralta R. Michel C. Vuilleumier P. (2005). Two electrophysiological stages of spatial orienting towards fearful faces: early temporo-parietal activation preceding gain control in extrastriate visual cortex. Neuroimage 26, 149–163. 10.1016/j.neuroimage.2005.01.015 15862215

[B64] Rapee R. M. Heimberg R. G. (1997). A cognitive-behavioral model of anxiety in social phobia. Behav. Res. Ther. 35, 741–756. 10.1016/S0005-7967(97)00022-3 9256517

[B65] Richards A. Blanchette I. Munjiza J. (2007). Contextual influences in the resolution of ambiguity in anxiety. Cogn. Emot. 21, 879–890. 10.1080/02699930601054018

[B66] Riwkes S. Goldstein A. Gilboa-Schechtman E. (2015). The temporal unfolding of face processing in social anxiety disorder—a MEG study. NeuroImage 7, 678–687. 10.1016/j.nicl.2014.11.002 25844308 PMC4377840

[B67] Rossignol M. Anselme C. Vermeulen N. Philippot P. Campanella S. (2007). Categorical perception of anger and disgust facial expression is affected by non-clinical social anxiety: an ERP study. Brain Res. 1132, 166–176. 10.1016/j.brainres.2006.11.036 17184747

[B68] Rossignol M. Campanella S. Bissot C. Philippot P. (2013). Fear of negative evaluation and attentional bias for facial expressions: an event-related study. Brain Cogn. 82, 344–352. 10.1016/j.bandc.2013.05.008 23811212

[B69] Rossignol M. Philippot P. Bissot C. Rigoulot S. Campanella S. (2012). Electrophysiological correlates of enhanced perceptual processes and attentional capture by emotional faces in social anxiety. Brain Res. 1460, 50–62. 10.1016/j.brainres.2012.04.034 22592075

[B70] Schultz L. T. Heimberg R. G. (2008). Attentional focus in social anxiety disorder: potential for interactive processes. Clin. Psychol. Rev. 28, 1206–1221. 10.1016/j.cpr.2008.04.003 18555570

[B71] Schulz C. Mothes-Lasch M. Straube T. (2013). Automatic neural processing of disorder-related stimuli in social anxiety disorder: faces and more. Front. Psychol. 4:282. 10.3389/fpsyg.2013.00282 23745116 PMC3662886

[B72] Schupp H. T. Ohman A. Junghöfer M. Weike A. I. Stockburger J. Hamm A. O. (2004). The facilitated processing of threatening faces: an ERP analysis. Emotion 4, 189–200. 10.1037/1528-3542.4.2.189 15222855

[B73] Schwarz K. A. Wieser M. J. Gerdes A. B. M. Mühlberger A. Pauli P. (2013). Why are you looking like that? How the context influences evaluation and processing of human faces. Soc. Cogn. Affect. Neurosci. 8, 438–445. 10.1093/scan/nss013 22287265 PMC3624952

[B74] Sewell C. Palermo R. Atkinson C. McArthur G. (2008). Anxiety and the neural processing of threat in faces. Neuroreport 19, 1339–1343. 10.1097/WNR.0b013e32830baadf 18695520

[B75] Spielberger C. D. Gorsuch R. L. Lushene R. E. (1970). State-Trait Anxiety Inventory. Palo Alto, CA: Consulting Psychologists Press.

[B76] Staugaard S. R. (2010). Threatening faces and social anxiety: a literature review. Clin. Psychol. Rev. 30, 669–690. 10.1016/j.cpr.2010.05.001 20554362

[B77] Straube T. Mentzel H. J. Miltner W. H. R. (2005). Common and distinct brain activation to threat and safety signals in social phobia. Neuropsychobiology 52, 163–168. 10.1159/000087987 16137995

[B78] Turner S. M. Beidel D. C. Dancu C. V. Stanley M. A. (1989). An empirically derived inventory to measure social fears and anxiety: the Social Phobia and Anxiety Inventory (SPAI). Psychol. Assess. 1, 35–40. 10.1037/1040-3590.1.1.35

[B79] van Peer J. M. Spinhoven P. Roelofs K. (2010). Psychophysiological evidence for cortisol-induced reduction in early bias for implicit social threat in social phobia. Psychoneuroendocrinology 35, 21–32. 10.1016/j.psyneuen.2009.09.012 19836898

[B80] Vuilleumier P. Armony J. L. Driver J. Dolan R. J. (2003). Distinct spatial frequency sensitivities for processing faces and emotional expressions. Nat. Neurosci. 6, 624–631. 10.1038/nn1057 12740580

[B81] Vuilleumier P. Pourtois G. (2007). Distributed and interactive brain mechanisms during emotion face perception: evidence from functional neuroimaging. Neuropsychologia 45, 174–194. 10.1016/j.neuropsychologia.2006.06.003 16854439

[B82] Vuilleumier P. Righart R. (2011). Attention and automaticity in processing facial expressions, in Oxford Handbook of Face Perception, eds Calder A. Rhodes G. Johnson M. Haxby J. (Oxford: Oxford University Press), 449–478.

[B83] Walentowska W. Wronka E. (2012). Trait anxiety and involuntary processing of facial emotions. Int. J. Psychophysiol. 85, 27–36. 10.1016/j.ijpsycho.2011.12.004 22210124

[B84] Watson D. Clark L. A. Tellegen A. (1988). Development and validation of brief measures of positive and negative affect: the PANAS scales. J. Pers. Soc. Psychol. 54, 1063–1070. 10.1037/0022-3514.54.6.1063 3397865

[B85] Weeks J. W. Howell A. N. (2014). Fear of positive evaluation: the neglected fear domain in social anxiety, in The Wiley Blackwell Handbook of Social Anxiety Disorder, ed Weeks J. W. (Oxford: Wiley-Blackwell), 433–453.

[B86] Wieser M. J. Brosch T. (2012). Faces in context: a review and systematization of contextual influences on affective face processing. Front. Psychol. 3:471. 10.3389/fpsyg.2012.00471 23130011 PMC3487423

[B87] Wieser M. J. Gerdes A. B. Büngel I. Schwarz K. A. Mühlberger A. Pauli P. (2014). Not so harmless anymore: how context impacts the perception and electrocortical processing of neutral faces. Neuroimage 92, 74–82. 10.1016/j.neuroimage.2014.01.022 24462933

[B88] Wieser M. J. Gerdes A. B. M. Greiner R. Reicherts P. Pauli P. (2012a). Tonic pain grabs attention, but leaves the processing of facial expressions intact - evidence from event-related brain potentials. Biol. Psychol. 90, 242–248. 10.1016/j.biopsycho.2012.03.019 22503790

[B89] Wieser M. J. Klupp E. Weyers P. Pauli P. Weise D. Zeller D.. (2012b). Reduced early visual emotion discrimination as an index of diminished emotion processing in Parkinson's disease? - Evidence from event-related brain potentials. Cortex 48, 1207–1217. 10.1016/j.cortex.2011.06.006 21764048

[B90] Wieser M. J. McTeague L. M. Keil A. (2011). Sustained preferential processing of social threat cues: bias without competition? J. Cogn. Neurosci. 23, 1973–1986. 10.1162/jocn.2010.21566 20807057 PMC3588162

[B91] Wieser M. J. McTeague L. M. Keil A. (2012c). Competition effects of threatening faces in social anxiety. Emotion 12, 1050–1060. 10.1037/a0027069 22390712 PMC3482481

[B92] Wieser M. J. Pauli P. Reicherts P. Mühlberger A. (2010). Don't look at me in anger! - Enhanced processing of angry faces in anticipation of public speaking. Psychophysiology 47, 241–280. 10.1111/j.1469-8986.2009.00938.x 20030758

[B93] Yoon K. L. Fitzgerald D. A. Angstadt M. McCarron R. A. Phan K. L. (2007). Amygdala reactivity to emotional faces at high and low intensity in generalized social phobia: a 4-tesla functional MRI study. Psychiatry Res. 154, 93–98. 10.1016/j.pscychresns.2006.05.004 17097275

[B94] Yoon K. L. Zinbarg R. E. (2008). Interpreting neutral faces as threatening is a default mode for socially anxious individuals. J. Abnorm. Psychol. 117, 680–685. 10.1037/0021-843X.117.3.680 18729619

