# Supplementary material for: The Effect of Affective Context on Visuocortical Processing of Neutral Faces in Social Anxiety
Source: Front Psychol. 2015 Nov 30;6:1824. doi: 10.3389/fpsyg.2015.01824 (PMC4663271; doi:10.3389/fpsyg.2015.01824)
Supplement: Supplementary file 1 [file Table1.DOCX]

**Supplementary Table 1**: The five items used in the pre-screening questionnaire (translated from German). Responses were obtained on a 5-point Likert scale (0 = “Strongly disagree” to 4= “Strongly agree”).

| Number | Item |
| --- | --- |
|  | I have a marked fear to be in social situations or performance situations and act in a way that will be embarrassing or humiliating. |
|  | Exposure to social situations or performance situations almost invariably provokes anxiety in me. |
|  | I recognize my fear of social situations or performance situations is excessive and unreasonable. |
|  | If possible, I avoid social situations and performance situations. If I cannot avoid them, I endure them with intensive anxiety or distress. |
|  | The avoidance of or distress in social situations and performance situations interferes with my normal routine. |
